# A Community Advisory Board’s Role in Disseminating Tai Chi Prime in African American and Latinx Communities: A Pragmatic Application of the Consolidated Framework for Implementation Research

**DOI:** 10.3390/healthcare13243307

**Published:** 2025-12-17

**Authors:** Ejura Yetunde Salihu, Kristine Hallisy, Jéssica S. Malta, Deborah Tolani Joseph, Cheryl Ferrill, Patricia Corrigan Culotti, Rebeca Heaton Juarez, Betty Chewning

**Affiliations:** 1 Social and Administrative Sciences Division, School of Pharmacy, University of Wisconsin-Madison, Madison, WI 53705, USA; betty.chewning@wisc.edu; 2 Department of Family Medicine and Community Health, University of Wisconsin-Madison, Madison, WI 53705, USA; hallisy@pt.wisc.edu; 3 School of Medicine and Public Health, University of Wisconsin-Madison, Madison, WI 53705, USA; malta.jessicas@gmail.com; 4 Department of Community Health, Hospital Sisters Health System, Springfield, IL 62707, USA; debbyfadex@gmail.com; 5 Inclusive Tai Chi Prime Community Advisory Board, Milwaukee, WI 53204, USA; cheryferr@gmail.com (C.F.); pat@enhancingbalance.com (P.C.C.); r.heatonjuarez@gmail.com (R.H.J.); 6 Enhancing Balance, Waukesha, WI 53189, USA

**Keywords:** community advisory board, community-based participatory research, Consolidated Framework for Implementation Research (CFIR), Tai Chi Prime, African American/Black, Latinx, fall prevention

## Abstract

**Background:** Community-Based Participatory Research (CBPR) has proven effective in promoting health research in hard-to-recruit and underserved populations. Tai Chi Prime is a National Council on Aging-certified fall prevention program. However, it has not been widely disseminated in African American (AA)/Black and Latinx communities. Guided by the Consolidated Framework for Implementation Research (CFIR), this study examined the process of working with a community advisory board (CAB) to adapt and disseminate Tai Chi Prime within these communities, as well as facilitators and barriers to CAB success. **Methods:** Eight CAB members met with researchers monthly virtually over a two-year period. Meetings focused on reviewing Tai Chi Prime materials, discussing cultural adaptations, and identifying dissemination strategies relevant to AA/Black and Latinx communities. Detailed notes from 24 meetings were compiled. In addition, semi-structured interviews were conducted with five CAB members and two researchers to capture individual reflections on their experiences, roles, and perceived impact. Data was analyzed using directed content analysis. **Results:** CFIR constructs helped illuminate how CAB members’ embedded community expertise, organizational partnerships, available resources, shared vision and transparent communication influenced the cultural adaptation and dissemination of Tai Chi Prime. Study findings also highlight important areas that extend beyond CFIR, particularly the cultural knowledge and power-sharing responsibilities undertaken by CAB members as co-researchers. These insights underscore the need to integrate equity-focused and community-engaged research principles into implementation frameworks when working with communities of color. **Conclusions:** Findings highlight the value of leveraging existing academic–community partnerships. Community-engaged researchers can use the lessons learned from this CAB to build a replicable model of sustainable partnerships with their AA/Black and Latinx community partners, as can others involved in health services research and policy.

## 1. Introduction

Community-Based Participatory Research (CBPR) is a community-driven approach for translating health research to real-world settings with the goal of improving individual and community health [1,2,3]. In CBPR, the intention is for community members to be active and empowered participants in research, giving input on every phase of the study, from design to dissemination. The equitable nature of CBPR makes it an appropriate model for working with vulnerable populations, including African American (AA)/Black and Latinx communities, both historically underserved populations in the U.S [4]. This research approach works for these cultural groups because it strengthens the research quality and improves the cultural relevance and fit. Studies have shown that health promotion programs that involve community members in the research process report better recruitment and retention rates, implementation, dissemination, and sustainability [4,5,6,7,8]. Studies also show that active involvement of community members in the research process significantly improves academic–community partnerships, builds trust, and translates to better health outcomes for the community [1,2,3,4,9].

Despite CBPR’s known benefits, navigating and minimizing power imbalance has been a consistent challenge in many CBPR studies. If left unchecked, this imbalance can lead to negative outcomes for program sustainability. Several authors posit that when researchers start out by singlehandedly deciding the research agenda, resolving conflicts, and leading decision-making, they establish a power hierarchy that is difficult to eliminate when the intervention has already been implemented [9,10,11,12,13]. To facilitate recruitment of AA/Black and Latinx individuals into health promotion programs, there is a need for replicable models to facilitate equitable community engagement and academic–community partnerships grounded in trust and shared leadership.

### 1.1. Community Advisory Boards

Community advisory boards (CABs) are an important component of CBPR. They provide a formal structure for involving community members in research activities and forging sustainable academic–community partnerships [6,14]. Several studies show that CABs facilitate health promotion research in hard-to-recruit and underserved populations because they are safe spaces for community members to have their voices heard and prioritized [2,6,15,16]. CABs are typically composed of community members, leaders, and/or staff of community organizations whose roles are to ensure that their communities’ needs are acknowledged and met, and that the research process is culturally respectful. To maximize the impact of CABs in research development and implementation, it is important that members are treated as partners rather than just advisors for the project [10,14,17,18]. Additionally, to meet the goals of CBPR, thoughtful, strategic planning is required when forming a CAB to ensure equitable partnership between the academic researchers and community partners [1,6,17].

### 1.2. Applying CBPR to Inclusive Tai Chi Prime in Communities of Color

Tai Chi Prime is a tai chi-based program approved by the National Council on Aging for fall prevention [19]. Tai Chi Prime builds on the Tai Chi Fundamentals-Adapted^®^ (TCF-A) Program, which was developed by Tricia Yu, founder of Tai Chi Health, LLC. TCF-A is a simplified and adaptive version of traditional Yang Style TC. TCF-A has seated and walker versions for people with limited mobility as well as optional side support (OSS) [19,20]. Unique to Tai Chi Prime is an embedded educational component with Practice Planners (goal setting) and Trackers (exercise accountability) to enhance home practice habits of participants.

A randomized controlled trial showed significant improvements in leg strength, gait/mobility, balance confidence, and cognitive function in participants who received Tai Chi Prime intervention compared to those in the waitlist control group [21]. Despite its proven efficacy for fall prevention and cognitive health, Tai Chi Prime has not been disseminated in AA/Black and Latinx communities. At the same time, there was a real question as to how relevant or attractive this Chinese tai chi exercise form, would be to AA/Black and Latinx communities. This study aimed to fill this gap by recruiting and training people from the target communities to offer Tai Chi Prime to their communities. Attempting to disseminate Tai Chi Prime in AA/Black and Latinx communities required thoughtful, careful planning with a CAB to ensure equitable power sharing between stakeholders, and culturally responsive adaptation while maintaining fidelity to the Tai Chi Prime curriculum.

This is the first CAB to adapt and disseminate Tai Chi Prime in communities of color. This qualitative study’s overall goal was to evaluate the embodiment of inclusivity to deliver Tai Chi Prime to these two communities in culturally meaningful ways by examining the process and outcomes of the CAB, including how they adapted and disseminated Tai Chi Prime in AA/Black and Latinx communities. This study also explored the barriers and facilitators of CAB engagement.

## 2. Materials and Methods

### 2.1. Study Sample

**Figure 1:**
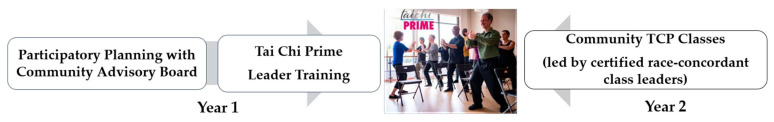
Project Timeline: Participatory Planning, Leader Training, and Community Tai Chi Prime Delivery.

In the spring of 2019, before submitting a grant for the Inclusive Tai Chi Prime study, the Principal Investigator (PI) and Co-PI asked for advice from the Community-Academic Aging Research Network (CAARN), a federally funded resource on campus which had relationships with community-based organizations serving AA/Black and Latinx communities in Milwaukee [22]. CAARN provides training and resources to researchers to facilitate engagement in community-based research. Based on their suggestions, the researchers initially invited two community-based organizations (one serving the AA/Black and the other serving the Latinx community) to participate in the study. These organizations were well respected in their communities, had experience writing grants, and had the potential to sustain Tai Chi Prime in their communities. They agreed to participate in early grant proposal conversations, and the resulting planning process reinforced the shared value and goal of inclusivity. These conversations shaped the details for grant activities including timing, space availability, budget commitments, and affirmed the goal of having members of the two communities be trained to become the Tai Chi Prime course leaders for their communities. To do this, there was an agreement they would undergo a year of training in the Tai Chi Prime leader pathway course in order to become certified. Figure 1 provides an overview of the project timeline, including planning with CAB, Tai Chi Prime leader pathway training for race-concordant leaders and subsequent delivery of Tai Chi Prime classes in the target communities.

When the proposal was funded, a third community-based organization serving AA/Black community was invited to join the CAB. These three community-based organizations helped identify other community members to join the CAB. The CAB started out with six members (two representing the AA/Black organizations, one representing the Latinx organization, two representing Tai Chi Health and one Tai Chi Prime Master Trainer). Four months into the project, each community partner nominated a community representative from the Tai Chi Prime leader training course cohort to serve on the CAB. These individuals helped ensure that the needs of the trainees and their communities were met, ensuring responsive course planning, recruitment, and delivery. These eight CAB members met with three university researchers monthly to plan, recruit and deliver Tai Chi Prime to the target communities. See Appendix A for an overview of the community organizations and partners that made up the CAB. All community members were paid $100 for each meeting to help compensate for their planning and overseeing each phase of the grant.

### 2.2. Data Collection

Two main data sources were used for this qualitative study: (1) CAB meeting notes and (2) interviews with CAB members and two researchers involved in the study.

CAB meetings occurred monthly between July 2022 and June 2024. Detailed minutes were recorded for each of the 24 CAB meetings. The PI took CAB meeting notes and shared them with the group at the end of each meeting. Agendas of the CAB meetings aligned with key decisions to be made at each phase of the grant: (1) recruitment of potential Tai Chi Prime course leaders from each community; (2) leader training schedule, place of training, refreshments; (3) adaptation of Tai Chi Prime delivery, e.g., translation of materials into Spanish; (4) certification of leaders’ readiness to teach; (5) selection of sites for community courses; (6) recruitment of community members to take the class; (7) evaluation of teaching fidelity; (8) participant evaluation of courses; (9) paying instructors.

To supplement the meeting notes, the lead author (an experienced qualitative researcher) conducted one-on-one semi-structured interviews with five CAB members and two researchers (Principal Investigator and Co-Principal Investigator). Interview guide questions aimed to capture individual reflections on their experiences, roles, and perceived impact on program outcome. A copy of the interview guide is provided in Appendix B. The interviews occurred via Zoom between August 2023 and April 2024. Each interview lasted 45–60 min. The interviews were audiotaped and transcribed.

### 2.3. Data Analysis

Three researchers (E.Y.S., J.M. and D.T.J.) with graduate-level training in qualitative research analyzed the CAB meeting notes and interview data using directed content analysis [23,24]. Data was managed and coded in NVivo software version 14 [25].

The researchers used deductive and inductive coding approaches to analyze the data [23,26]. The Consolidated Framework for Implementation Research (CFIR) constructs: innovation (e.g., adaptability and evidence-base), individuals (e.g., characteristics and roles), inner setting (e.g., communications and available resources), outer setting (e.g., financing, partnerships, and connections), and implementation process (e.g., adapting, and reflecting and evaluating) formed the initial categorization matrix and was useful for interpreting the data and reporting the findings [27,28]. The research team first immersed themselves in the data through repeated readings of the transcripts and CAB meeting notes, then applied the CFIR-informed coding matrix to guide deductive coding. Data segments that did not align with predetermined codes were coded inductively and added to the matrix as emergent codes. Both deductive and inductive codes were consolidated to create categories then organized into themes through an iterative process [23,24]. Data collection and analysis for the CAB meeting notes and interviews occurred simultaneously.

Lincoln and Guba’s criteria for ensuring rigor in qualitative research (credibility, dependability, confirmability, and transferability) was employed [29,30,31,32]. Investigator triangulation (multiple coders), data triangulation (meeting notes and interview data), peer debriefing and member checking supported credibility. The lead author maintained a reflexive journal to document her assumptions, analytic decisions, and evolving interpretations, enhancing confirmability and dependability. Detailed descriptions of participants and research context have been provided to support transferability. The research team comprises individuals with diverse academic backgrounds and skillsets, including community-based research, implementation science, cultural adaptation, and qualitative methods, which supported the study design and data interpretation.

## 3. Results

Eight CAB members and three researchers attended the monthly CAB meetings. All CAB members identified as female and college educated. Each CAB meeting recorded a mean attendance of six of the eight CAB members. The high attendance reflects the high engagement of CAB members throughout this study.

This section presents themes and quotes related to each of the CFIR constructs and elucidates the process that led to CAB outcomes. Quotes are cited as a specific CAB member (CAB 1) to designate the person interviewed. When the source is from the monthly Advisory Board minutes (ABM#12), the number of the meeting is designated at the end of the quote. Table 1 provides an overview of CFIR domains, constructs and resulting themes captured at each stage of project.

### 3.1. CAB Formation and Research Context

This stage captures the contextual factors that shaped the development of the CAB and the environment in which the Inclusive Tai Chi Prime project was launched. Analysis of this stage drew primarily on CFIR’s Individuals and Innovation domains to understand the characteristics of CAB members, their roles, their relationships with each other, and their perceptions of the Tai Chi Prime program. The following CFIR domains, constructs and emerging themes illustrate how CAB member characteristics and Tai Chi Prime design informed early project planning and program fit in the target communities.

#### 3.1.1. CFIR Domain 1: Individuals

This domain captures the characteristics of CAB members (including their roles in their organizations, prior experiences, and existing relationship within and outside their communities) and the roles they played in the program.

Construct 1: CAB Members Characteristics

CAB members’ long-standing involvement in their communities, their professional expertise, and their existing relationships with other community-based organizations shaped how they engaged with the program and supported its activities. See Appendix A for an overview of the community organizations and partners that made up the CAB.

Theme 1: CAB members and their organizations are embedded and respected in the communities they serve.

CAB members were regarded as respected community leaders. The organizations they represented also played a long-standing and trusted role in the target communities. CAB members described their work as deeply rooted in community empowerment. This embeddedness in the target communities strengthened their ability to engage community members, identify local needs, and serve as credible messengers that faciliated Tai Chi Prime delivery. As one CAB member explained: “Our work [focuses] on connecting community health workers to underserved populations to facilitate care coordination.” CAB 3.

Similarly, another CAB member said: “…[I joined this project because of] my role as a community health building manager…we had direct access to residents in the community.”—CAB 4.

Theme 2: Pre-existing relationship between CAB Members’ organizations.

Several members described how introductions through established professional networks created natural pathways for participation and strengthened early buy-in. For some, involvement in the CAB emerged directly from ongoing partnerships with other members.

One CAB member described how an existing connection with a fellow CAB member created an unexpected but welcomed opportunity to join the project: “CAB 8 introduced me to the Tai Chi Health organization. And I started helping her with seminars and workshops, and even her classes. And here’s a funny story. On Facebook, I saw an ad to start teaching Tai Chi in Spanish, and I was like, ‘Hey, I know Spanish, I know tai chi, I can help out.’ I had no idea that CAB 8 was part of the program. I sent out my information to CAB 1, and I saw CAB 8’s name so I contacted her, and she said, ‘Oh, you want to be part of this project.’ I said, ‘Absolutely,’ and then I became involved in the project.”—CAB 7.

Another CAB member had a similar experience: “I was actually introduced to the project after working with CAB 3… I’m really thankful to CAB 3 for bringing me on board.”—CAB 4.

Construct 2: CAB Members’ Roles

This construct captures the key roles that CAB members played to ensure the successful planning and delivery of the Tai Chi Prime in their communities.

Theme 1: CAB members ensured cultural fit of program.

CAB members ensured that the program addressed the specific needs of AA/Black and Latinx communities and that instructional materials were culturally relevant and inclusive. As one CAB noted: “…my role as an advisory board member was to make sure that equity and inclusion was included in the program. We kept the needs of the residents and folks participating in the instructor training first, and we made sure that we spoke to each community in a way that was culturally relevant.”—CAB 4.

Theme 2: CAB members supported program logistics and program delivery.

CAB members supported program delivery by assisting with “some outside tasks related to preparation for meetings and/or emails related to ongoing communication that relates to the work of this board.”—ABM #5—November 2022.

They helped organize the TCP leader pathway classes and handling some of the logistics, as agreed in ABM #6 (December 2022): “CAB 4 will order a Mindful Lunch, arrange pick up/delivery and set-up with CAB 1 on Day 1 (January 26) at noon” and ABM #7 (January 2023): “CAB 7 will provide some brewed teas and the all-important snack—chocolate!”

#### 3.1.2. CFIR Domain 2: Innovation

This construct focuses on the perceived benefits of Tai Chi Prime for the target communities and adaptable elements of Tai Chi Prime delivery, including what makes this evidence-based program suitable for them.

Construct 1: Innovation Evidence-Based

CAB members believed that Tai Chi Prime is a multifaceted health promotion program that could reduce fall risk, and improve mental and emotional well-being in all age groups.

Theme 1: Existing evidence supporting Tai Chi Prime as a fall prevention program and potential for other health benefits.

CAB members believed that the additional benefits of Tai Chi Prime practice, besides being an evidence-based fall prevention program, was a significant factor that contributed to the high acceptance within the community.

CAB members from both communities emphasized the importance of highlighting the health benefits of tai chi beyond Tai Chi Prime’s fall prevention, balance and cognition benefits, as health factors such as overall well-being, mental health and stress management were relevant to their communities.”I know that the research was based on balance and prevention of falls for aging and older adults, which is necessary, especially with the growing elderly population but we have a community that includes youth that could benefit from the mental health aspects of Tai Chi…. The feedback that we got repeatedly was that they [other community members] wanted it for the youth. They wanted it for their grandkids.”—CAB 4.

Another CAB member highlighted how their organization was actively trying to integrate Tai Chi Prime into broader community and county-level discussions, emphasizing its perceived importance and potential impact: “…we have actually included this in our dialogue with Milwaukee County Department on Aging because we know it’s important for this program to exist.”—CAB 3.

Construct 2: Innovation Adaptability

This construct addresses the adaptable elements of Tai Chi Prime, and the implications of adapting the program delivery for different cultural groups.

Theme 1: Tai Chi Prime’s history reflects its adaptability for different cultural contexts.

CAB 8, an experienced master trainer who taught and certified the AA/Black and Latinx leader trainees through the entire Tai Chi Prime leader pathway course, believed that linguistic adaptations were urgently needed for the Latinx community to facilitate Tai Chi Prime uptake by both leader trainees and community participants. CAB 8 also believed that given the Chinese origins of tai chi there is proof that the language and cultural presentation of TC is highly adaptable to meet the needs of other cultural groups.

“Tai chi came from a different culture as well…So, that kind of gets you over that hurdle right at the get go because it’s [tai chi] always been trying to bridge the cultural gap.”

—CAB 8.

### 3.2. CAB Process

This stage focuses on the contextual factors that supported CAB engagement and Tai Chi Prime implementation in the target communities. Guided by CFIR’s Outer Setting and Inner Setting domains, we examined how external partnerships, communication, organizational resources, and funding considerations influenced the CAB’s functioning.

#### 3.2.1. CFIR Domain 3: Outer Setting

The outer setting domain covers external factors and resources that were employed to facilitate the CAB process, including Tai Chi Prime adaptation and dissemination.

Construct 1: Partnerships and Connections

This construct covers external partnerhsips and connections that the CAB leveraged to meet the needs of the program.

Theme 1: Leveraging partnerships and resources from other institutions.

CAB members leveraged their existing partnerships with other institutions to meet some of the program’s needs.

“…we often host two interns in the first semester of the [university] school year. This year, the two assigned students did an amazing job just laying the landscape for places to share literature about tai chi. They did some mapping to see where senior housing is or was located and identified laundromats and grocery stores that those individuals may use as good marketing places for tai chi, so that was really good work done internally by those team members.”

—CAB 3.

“We are also currently seeking funding from some of our already established funders whose values and missions align with the work of the Tai Chi Prime program.”

—CAB 4.

Construct 2: Financing

This construct addresses the availability of funds from external sources such as grants to support CAB engagement and program delivery, as well as the financial challenges that arose during the course of the program.

Theme 1: Navigating funding constraints through collaborative problem-solving.

This study received funding from three institutional sources, which supported key activities such as compensating CAB members, securing sites for training Tai Chi Prime leaders, and providing refreshments during community Tai Chi Prime classes. Although the available funding helped launch and sustain study activities, some finance-related challenges emerged during implementation that required the team to engage in collaborative and creative problem-solving. For example, when the CAB decided to film each race/ethnic cohort of the Tai Chi Prime leader training for promotional use, the cost of a professional photoshoot exceeded the budget, so a CAB member did the photoshoot at a discounted rate.

As one team member noted during an advisory board meeting: “[The research team] has discussed a few places where we could trim some budget, but this might take some creativity from all of us.”—ABM #0—June 2022.

This underscores how fiscal constraints prompted strategic adjustments while maintaining the integrity of the program’s goals.

#### 3.2.2. CFIR Domain 4: Inner Setting(s)

The Inner Setting construct covers internal factors and strategies, such as communication and available resources, that were employed within the group to facilitate the CAB process.

Construct 1: Communication

This construct covers the type and frequency of communication that was employed by the CAB to ensure engagement, share updates, and make collective decisions.

Theme 1: Monthly CAB meetings to ensure consistent communication.

The team convened monthly for an hour-long meeting held via virtual paltform (Zoom). Meetings were facilitated by the study’s PI. The meeting day and duration were decided by consensus among the team members. CAARN assisted with setting up Zoom links for each meeting. Agendas for each meeting were determined by the stage and need of the grant and communities.

“Block personal/agency calendars to secure regular attendance of AB1. PI to release meeting agenda out to the team 7–10 days prior to the next meeting.”

—ABM #0 (kick-off meeting) by Study PI.

Construct 2: Available Resources

This constructs focuses on the availability of resources within the organizations represented in the CAB to support Tai Chi Prime delivery. Discussions highlighted how resource availability influenced the team’s ability to plan, implement, and sustain program activities.

Theme 1: Leveraging organization resources to support program delivery.

Several CAB members offered their organization facilities and resources to support program delivery. For instance, a CAB member offered her organization’s community center as a possible space for hosting both in-person and virtual training, which ensured flexibility in training delivery.

“CORE has a room with mirrors and AV availability if we need to Zoom to virtual participants (although in-person training is preferred). Space needs: 6’ × 6’ per person = 36 sq ft/person. A 1000–1500 sq ft space could allow for 27–41 persons.”

—ABM #1—July 2022.

### 3.3. CAB Outcomes

This stage reflects the tangible outcomes that emerged from CAB engagement. Analysis drew on CFIR’s Implementation domain (with a focus on adapting, and reflection and evaluation constructs) to understand how the CAB shaped delivery of Tai Chi Prime in culturally relevant ways and how members assessed their own participation, highlighting perceived facilitators and barriers to their engagement.

#### 3.3.1. CFIR Domain 5: Implementation Process

**Figure 2:**
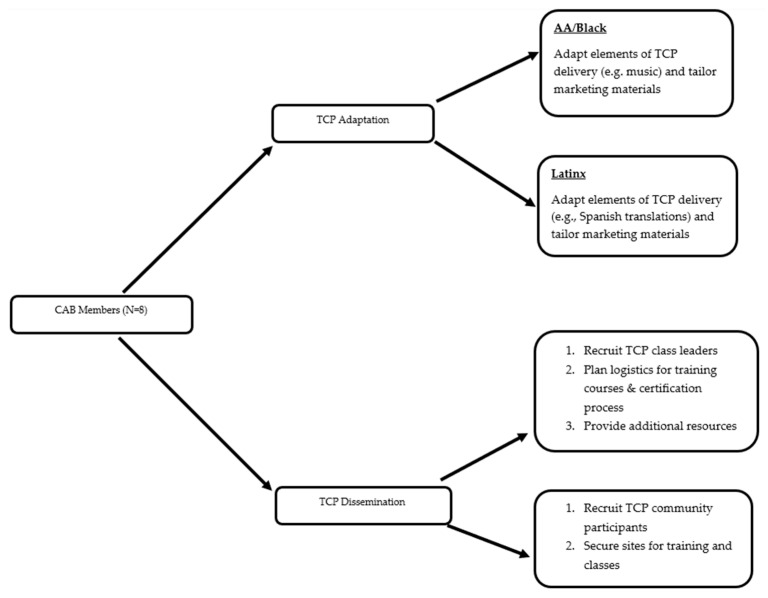
CAB’s role adapting and disseminating Tai Chi Prime in AA/Black and Latinx communities.

The Implementation construct covers how the CAB meet their objective to adapt and disseminate Tai Chi Prime in AA/Black and Latinx communities, as well as the strategies they employed to achieve these outcomes (See Figure 2).

Construct 1: Adaptation of Tai Chi Prime delivery

This construct addresses the types of adaptations the CAB made so that delivery of the Tai Chi Prime optimally fit the target communities, and the rationale for these changes.

Theme 1: General tailoring of marketing materials to meet communities’ needs.

Previous Tai Chi Prime research had developed single-page and two-page documents, as well as educational PowerPoint marketing tools to advertise the program and its health benefits. CAB members were free to adapt and/or develop their own marketing and enrollment strategies to be more suitable for their communities.

“We needed a promotional video to help us with this messaging, to give us more of a visual. We were able to take some of the [existing] templates and change out some pictures to be a little more local to the communities that were being presented…I thought that was wonderful.”

—CAB 3.

To support this, funds were allocated for a photo shoot. With CAB 8’s supervision, the team ensured fidelity to Tai Chi Prime postures in the marketing photos and videos. The goal was to produce images featuring newly certified Tai Chi Prime class leaders and community sites where future Tai Chi Prime courses will be offered, providing culturally relevant and engaging advertising materials that resonate with each community.

Theme 2: Adaptation of Tai Chi Prime delivery for Latinx community.

A Latinx-specific subcommittee was formed, with CAB 7 leading the group. Three Latinx individuals who were enrolled in the Tai Chi Prime leader pathway training courses also volunteered to join the group. The group met weekly for nine months to work on the Spanish translations. The group often met for one hour once a week but started meeting three times a week as they approached the deadline. Their task was primarily to support the work of a professional translator in translating selected Tai Chi Prime materials to Spanish. The team reviewed translated sections of the curriculum and handouts for Latinx class leaders. Their goal was to ensure that the original meanings of the Tai Chi terminologies were not lost in translation:

“…when it comes to Tai Chi, there are certain terminologies that have to be changed to represent what Tai Chi principles are. So, you can’t be literal with some of the translations. That is one constraint when it comes to translation. The second constraint is we’re trying to appeal to the Spanish community, and the language is a little different. So, if they tried to translate it for Spanish people from Spain, it wouldn’t connect with people who are more Central American …We have a different type of linguistics and language. So, I thank God for [other group members], because they’re very good at that. We’ve had many discussions on how to change different things, in different contexts of the text.”

—CAB 7.

The team composition also improved their efficiency. Three of the members had stronger ties to the Latinx communities, having recently arrived in the United States and were more familiar with colloquial conversations in Spanish.

“I think our advantage is that [the other team members] are fairly new to the country, so they’re more connected to the Spanish communities. They haven’t lived in America for too long, and I have been living away from South America for over 20 years. So, I sometimes feel like I’m forgetting my Spanish and the culture, but they are more connected to the culture than I am…they know not just slang words, but more colloquial types of communication in Spanish. And because the majority of Spanish speaking people here in Wisconsin are from Mexico, I think it comes in handy for sure.”

—CAB 7.

Final changes, including Spanish translations of instructional materials, marketing and recruitment materials, Tai Chi Prime fillable PowerPoint slides, and handouts, were made to ensure accessibility for Spanish-speaking participants.

“Marketing materials were provided in Spanish, so it was very helpful just to have something as a guide, and now they have handouts in Spanish…I think it provides a lot of comfort to the instructors knowing that they have resources that they can provide their students.”

—CAB 1.

Theme 3: Adaptation of Tai Chi Prime delivery for AA/Black community.

An AA/Black-specific subcommittee was also formed, with CAB 2, CAB 3 and CAB 4 as members. The group discussed adapting certain elements of Tai Chi Prime, such as marketing materials and music choice.

The AA/Black subcommittee made changes to the promotional video and other marketing materials to appeal to their community.

“The AA/Black Tai Chi Prime team is interested in a brief video to sell the nuances of the TCP program to their communities… [The AA/Black Tai Chi Prime team] is looking for a 1 to 2-min video with a voice over specific to the AA/Black community with tai chi demonstration including modifications going on in the background.”

—ABM #12—June 2023.

The AA/Black subcommittee also discussed the potential use of culturally relevant music to enhance their participants’ engagement with the sessions and overall comfort.

“A rich discussion ensued about adaptations that may be needed for the African American community. CAB 5 stated that their tai chi practice group that has been meeting on Tuesdays and Friday nights explored using music (Miles Davis jazz) when doing basic moves training as a way of forming a cultural connection specific to the African American/black community.”

—ABM #7—January 2023

To stay true to tai chi principles, the team agreed to the following:

“Music is a wonderful cultural adaptation that the Tai Chi Prime team feels both communities can readily explore and use with a few stipulations: (1) Don’t use music early in tai chi training as it can create cognitive (mental) overload when first learning the kinesthetic (movement) demands of tai chi; (2) it is best if used in summative sessions of silent guided Basic Moves or Form practice sessions, (3) Avoid music with any strong rhythm that might turn tai chi into a dance! Finally, CAB 8 brought up the issue of music rights which limits the ability of using musical recordings in public spaces.”

—ABM #8—January 2023.

Theme 4: Adaptations made to the Tai Chi Prime leader certification process.

CAB members recognized the need to adapt the certification process to accommodate participants’ diverse backgrounds and varying comfort levels, particularly concerning the written tests.

“Normally, people take a written exam and you just send them a PDF and they’re on their own to figure out how to fill it out and what to do, and so what we made was an adaptation. We took each question and put it on a single page and then said first you outline, then you describe, then you say in your own words, you know, summarize. So, the written exams were designed to help people have the language they need when they teach. We templated all those questions for them and gave them a couple of examples from the ROM [Tai Chi Health’s Range of Motion] dance, and then I gave them answers. This way, they would understand what we were looking for… we had no idea of the diversity of people we were attracting to train and where they were, from a comfort level, in dealing with written tests, and some of these folks were educated. They have master’s and bachelor’s degrees, but if they are older, they are out of school for years, right? So, we wanted to make that adaptation to help them feel comfortable doing written tests.”

—PI.

#### 3.3.2. Construct 2: Dissemination of Tai Chi Prime

The second key objective of the CAB was to disseminate the adapted Tai Chi Prime in their respective communities. One way they did that was to assist with planning, recruitment and delivery of the Tai Chi Prime leader training courses.

Theme 1: Recruitment of Tai Chi Prime class leaders.

CAB members used several recruitment strategies to attract people to the Tai Chi Prime leader training courses held between Fall 2022 and Spring 2023. They advertised the Tai Chi Prime leader training courses through their network of community health workers.

“We supported in helping to identify community participants and facilitated the information through our network for community health workers… We had monthly check-ins with our workforce of community health workers to let them know about those opportunities, and we spoke at some churches that were being considered as host sites.”

—CAB 3.

CAB members also connected with wellness practitioners at resident engagement meetings. One CAB member who used this approach stated: “We connected with residents who were wellness practitioners. We introduced the program at the resident engagement meeting that happens every Tuesday, and CAB 8 showed the [promotional] video, and spoke with them about it…we knew which residents we wanted to engage and be part of it because of our asset inventory.”—CAB 4.

The Co-PI confirmed the reports of the CAB members. She said: “They used their networks of what I call the body people; people who are already doing yoga, maybe doing tai chi, meditation, dancing, Pilates even. I think they reached out to people who were already kind of trainers in their own way, more than anything else. They did a good job.”—Co-PI.

Following the CAB’s recruitment efforts, thirty-nine people expressed interest in registering for the Tai Chi Prime leader training courses. Twenty-six candidates (13 Latinx, 12 AA/Blacks, and one Indigenous American Indian) were admitted to the Tai Chi Prime leader training pathway. Thirteen individuals had to be placed on a waitlist due to limited space and funding. This exceeded the original recruitment goal (N = 10 per community). Nineteen individuals completed the required 30 contact hours to become certified class leaders. Eleven (n = 6 AA/Black and n = 5 Latinx) are now certified Tai Chi Prime class leaders. Details on the acceptability and feasibility of the Tai Chi Prime leader pathway training, along with fidelity assessments of the newly trained leaders who delivered the community Tai Chi Prime classes are presented in another paper [33].

Theme 2: Recruitment of community Tai Chi Prime participants.

After the candidates were certified and ready to lead community classes, CAB members recruited participants for these community Tai Chi Prime classes. They employed diverse marketing channels such as social media, websites, flyers at community sites, and discussions with potential host sites. Six community classes were delivered (three in AA/Black and three in Latinx community) across four community centers and one clinic in a 9-month period.

Recruitment and retention rates for the community Tai Chi Prime classes also exceeded the original goal (n = 10 per class or 60 people in total). One-hundred and forty-four individuals attended at least one of the classes offered in the communities (AA/Black, N = 58 and Latinx, N = 86). It is worth noting that the clinic site used a different model from the other community centers. In the clinic model, participants were referred to the Tai Chi Prime class from the diabetes clinic by their healthcare providers. Instead of the original twice a week 6-week format, classes were held once a week for 12 weeks.

**Figure 3:**
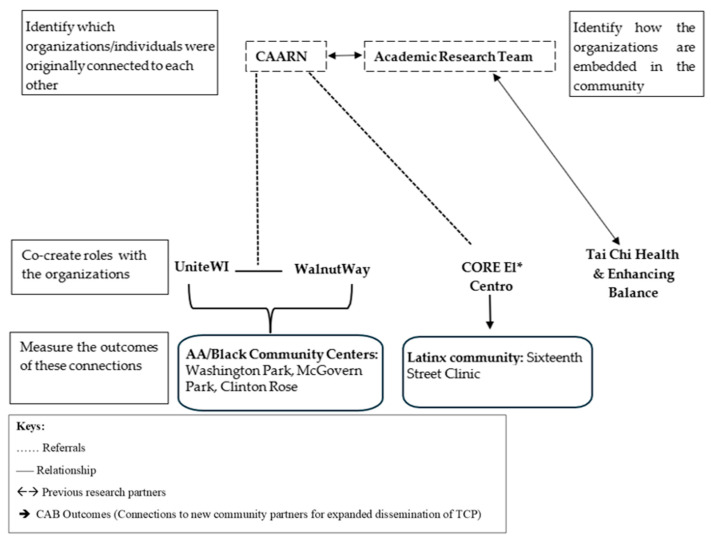
Stakeholder network mapping of CAB organizations: From initial connections to established partnerships. * Two community TCP classes were held at CORE El Centro.

Figure 3 illustrates how the academic research team strategically built relationships with AA/Black and Latinx leaders and community-based organizations and how these relationships led to dissemination of Tai Chi Prime in these communities.

#### 3.3.3. Construct 3: Reflection and Evaluation

CFIR’s reflection and evaluation construct was used to explore CAB members’ assessment of the CAB process and outcomes. This construct captures members’ reflections on what supported or hindered their participation, how they perceived their contributions, and the factors that shaped their overall experience within the CAB. Table 2 below provides an overview of the reflections of CAB members on the barriers and facilitators of CAB engagement and outcomes.

## 4. Discussion

This study examined how the CAB supported the cultural adaptation and dissemination of Tai Chi Prime in AA/Black and Latinx communities. Guided by CFIR, the findings demonstrate how multi-level implementation determinants, including individuals, inner and outer settings, and implementation processes, have influenced Tai Chi Prime delivery and sustainability in two culturally distinct communities. The data also revealed areas that extended or challenged existing CFIR domains, offering insights into how the framework can be expanded for community-engaged implementation research. Following recommendations by Kirk et al. [28], we describe how we applied CFIR at various phases of implementation from pre-implementation/planning (CAB formation) to implementation (process) and evaluation (outcomes). We also provide a clear linkage between CFIR constructs and the implementation outcomes.

### 4.1. CAB Formation and Research Context: Individual Characteristics and Innovation Attributes

We used the CFIR’s Individuals domain to examine the early formation of the CAB. We found that CAB members had several years of experience in grassroot mobilization, and were deeply embedded and respected within their communities, which aligns with CFIR’s focus on individuals’ characteristics (i.e., high-level leaders, opinion leaders, capability, and motivation) [27,28]. We also realized that CAB members contributed more than individual characteristics; they represented organizations with strong ties with their communities and strong sense of responsibility to promote community well-being, blurring the line between “individual,” “inner setting (e.g., culture)” and “outer setting (e.g., local attitudes).” This highlights a limitation with CFIR in capturing the fluid overlap between individual-level credibility, organizational legitimacy and socio-cultural values, three factors that facilitated program delivery and reach.

Given that this study involved two distinct communities, it was important that CAB members were representative of their respective communities and had a history of working together. The diverse recruitment strategies employed to recruit CAB members (referrals from previous partnerships, word of mouth, etc.) ensured that the group was representative of the target communities. This approach is supported by recommendations that community members recruited to join the CAB be broadly representative of their target communities [10,13,14,15,34,35,36]. By inviting community members enrolled in the Tai Chi Prime leader training to the CAB, we gained new perspectives on the feasibility of proposed adaptations to leader training courses and program delivery. This collaborative approach to training future Tai Chi Prime leaders was well-received, with the newly trained leaders rating their training as highly satisfactory [33].

Another unique feature of this board’s composition was that we were able to compare and contrast, in real time, initial reactions to the adaptations being made by AA/Black and Latinx community leaders. This led to deep and rich discussions that might have been otherwise missed if we formed separate CABs for each community. Additionally, CAB members were female, college-educated, and many had prior relationships with one another before this study. There was strong engagement within the group, which was sustained throughout the study. Another study found similar results which they attributed to their shared identities and experiences [37]. Despite the multicultural representation within our CAB, members leveraged their shared experiences and rapport, which led to better collaboration and decision-making. At the time of writing this manuscript, CAB members had begun combining resources and collaborating to apply for local grants to fund future Tai Chi Prime classes in their communities. This study, therefore, reinforces the value of exploring strategies to recruit individuals with commonalities, such as a deep commitment to serving their communities, when forming a CAB [38,39,40].

Within the Innovation Characteristics domain, CFIR constructs such as evidence-base and adaptability helped contextualize how CAB members perceived Tai Chi Prime. CAB members recognized Tai Chi Prime as an evidence-based intervention for reducing fall risks, which led to initial stakeholder buy-in and program uptake. In addition, participants’ belief in the program’s efficacy in improving mental health across diverse populations, including youth, strengthened their resolve to make the program sustainable. Given that a recent systematic review and meta-analysis demonstrated that tai chi-based interventions have positive impact on the physical and mental health outcomes in young people [41], future studies should investigate the potential of Tai Chi Prime and related programs in youth populations. Additionally, tai chi-based exercises have a long history of adaptation across diverse cultural contexts [42], which reinforced CAB members’ willingness to adapt Tai Chi Prime delivery for AA/Black and Latinx older adults. These findings highlight the importance of cultural resonance as an adaptation driver [43].

### 4.2. CAB Processes: Communication, Resources, and External Partnerships

We used CFIR’s Outer Setting domain to examine how external factors and partnerships shaped the CAB’s functioning. CAB members leveraged their existing relationships with other community-based organizations and local wellness service providers to support recruitment. Findings also revealed forms of resource sharing and informal support networks among community partners that extend beyond CFIR’s typical description of external partnerships. Community members shared space, staff time, transportation, cultural materials, and interpersonal labor in ways that reflect the relational fabric of underserved communities. This form of organic and reciprocal collaboration is not explicitly captured in CFIR. CAB members’ collaborative strategies for navigating funding constraints further illustrated the limitations in accounting for “real-world” resource environments. While CFIR acknowledges the role of financing (as a construct in outer setting e.g., grants) and funding (as a construct in inner setting e.g., time and space), it does not account for how both factors are often intertwined in community-based organizations that solely depend on external funding to support their internal processes and programming. The chronic systemic factors that characterize community-based organizations such as persistent underfunding, understaffing and high staff turnover [37,44] or the creative problem-solving these organizations employ to navigate these financial challenges are also not adequately captured in current implementation science literature.

The Inner Setting domain (i.e., communication and available resources), was also vital in explaining how CAB processes unfolded and how they influenced outcomes. Monthly meetings and a culture of mutual respect strengthened the CAB’s ability to make collective decisions and address challenges efficiently. Importantly, there was strong alignment between the research goals, and the mission and culture of the organizations represented on the CAB. This “mission alignment,” which is a core CFIR construct, supported collaboration, fostered trust, and contributed directly to the adaptation and dissemination of Tai Chi Prime. The values (i.e., community empowerment, wellness promotion, and equity-oriented service) that characterized the CAB members and their organizations created an environment where Tai Chi Prime could be meaningfully tailored, implemented and sustained.

### 4.3. CAB Outcomes: Adaptation and Dissemination

CFIR’s Implementation Process domain was used to examine CAB outcomes, including Tai Chi Prime adaptation and dissemination. CAB members tailored marketing materials, adapted delivery approaches (including language translation and culturally resonant music integration), and refined certification requirements to ensure fit for AA/Black and Latinx communities. These adaptations reflect deep cultural tailoring and reveal the limitations of traditional implementation science language in capturing culturally grounded adaptation work led by community partners rather than researchers [45]. Factors that influenced recruitment of class leaders and community participants, and program adaptation were interpreted through CFIR’s emphasis on planning and tailoring strategies, respectively. The recruitment challenges described by CAB members (such as initial community reluctance due to religious reasons and limited organizational resources) have been noted in prior studies [38,39]. These challenges suggest that there is an opportunity for community-engaged researchers to integrate concepts such as historical mistrust, community labor burden, and resource constraints when using CFIR.

### 4.4. Extending CFIR for Community-Engaged Implementation

CFIR constructs were helpful for exploring the facilitators and barriers to CAB outcomes. Common facilitators included acknowledgment of CAB expertise and co-ownership of project, alignment of community and research goals, and additional resources beyond the study period to ensure program sustainability. Barriers that were noted include insufficient funds and staffing to support all aspects of program implementation, and a complex payment system. These factors aligned with CFIR but also highlight areas of community-led work that CFIR does not fully address.

Based on this study’s findings, we suggest that community-engaged researchers account for these factors when using CFIR or similar implementation frameworks: (1) the interplay of organizational legitimacy within the community they serve, socio-cultural values and individual credibility; (2) cultural adaptation as a core implementation strategy; (3) the unique labor and resource constraints that community-based organizations face; (4) navigating historical mistrust and community hesitancy in early recruitment; and (5) the importance of acknowledging community members as both intervention recipients and implementation partners. These suggestions reinforce calls within the implementation science field to adapt CFIR for equity-centered, community-engaged work, where power-sharing, cultural fit, and structural constraints are key determinants rather than peripheral factors [45]. These recommendations also validate work by other researchers who tailored CFIR constructs to better match their research aims and population needs. For example, one study adapted the CFIR constructs to better suit their implementation context by delineating the multiple layers of a health system in the inner setting domain and emphasized patient needs as a domain rather than a subconstruct in their analysis [46]. Another study demonstrated that integrating race-specific considerations into CFIR constructs improved the identification of contextual barriers and facilitators influencing intervention processes and uptake, factors that would have been missed if CFIR was only deployed as a race-neutral tool [47].

An important question that emerged while preparing this manuscript was, “How do we adequately acknowledge and appreciate the unique expertise and significant work that our community partners did as co-researchers?” Based on previous research and our findings in this study, it is clear that CABs play a crucial role in adapting and disseminating programs within communities of color. They have a wealth of knowledge and lived experiences that ensure interventions are culturally relevant, grounded in real-world experience, and address the unique needs of the communities, all of which go beyond the traditional role of research participants. CAB members also invest significant time, emotional labor, and resources to attend meetings. In line with CBPR principles, the research team intentionally sought to elevate CAB members’ contributions in ways that recognize both their labor and their intellectual ownership of the work [36,48]. We have included CAB members’ backgrounds and perspectives in this report and ensured adequate compensation for their engagement. CAB members also reviewed final products, such as conference posters, grant reports, and academic publications, and are co-authors on publications [33].

Overall, this study challenged the traditional application of CFIR by intentionally engaging CAB members as representatives of the target population, implementation champions and recipients of the intervention from grant writing phase to program implementation and dissemination of findings. This approach directly responds to calls within the implementation science field to meaningfully involve recipients in CFIR-informed data collection and analysis [45,49,50]. Rather than relying on organizational decision-makers’ perceptions of recipients’ needs, we engaged the innovation recipients as implementation partners with legitimate influence and power. Findings demonstrate that recipients offered rich, contextually grounded insights that would likely be overlooked in a traditional, top-down application of CFIR.

This study has some limitations. First, not all CAB members were available to participate in the interviews due to conflicting schedules. As a result, some insights into group dynamics or individual experiences may not have been fully represented in this paper. Additionally, these findings are based on data from a single CAB, which may limit generalizability to other community contexts. Despite these limitations, the combination of CAB meeting notes and interview data provided an in-depth examination of the CAB process and factors that influenced the outcomes.

## 5. Conclusions

Using CFIR as an analytic lens was useful in examining this CAB-led Tai Chi Prime adaptation and dissemination process, and the limitations of traditional implementation frameworks in fully capturing the realities of community-engaged research. The success of the CAB highlights the importance of equitable academic–community partnerships in enhancing the relevance and reach of health promotion programs, such as Tai Chi Prime in underserved communities of color. Community-engaged researchers can use the lessons learned from this CAB to build a replicable model for developing community capacity and increasing access to health promotion and wellness programs, in communities of color. These insights could also be used to refine implementation frameworks like CFIR to better account for the complexities of community-engaged research. As we shift to more community-driven, inclusive health research where communities have a “seat at the table,” researchers are encouraged to continue to consider the plethora of meaningful ways of appreciating CABs, including providing equitable compensation, co-authorship on papers where appropriate, and using language that reflects the status of community partners as *active* and *equal partners* in research. This approach ensures that CAB members are fully acknowledged and respected for their work in moving implementation research forward.

## Figures and Tables

**Table 1:** Overview of CFIR domains, constructs and resulting themes mapped to project stages.

Project Stage	CFIR Domains	Subdomains/Constructs	Themes
Cab Formation and Research Context	Individuals	CAB Members’ Characteristics	CAB Members’ organizations are embedded and respected in the communities they serve
			Pre-existing relationship between CAB members’ organizations
		CAB Members Roles	CAB members ensured cultural fit of the program
			CAB members supported program logistics and program delivery
	Innovation	Evidence-Base	Existing evidence supporting Tai Chi Prime as a fall prevention program and potential for other health benefits
		Adaptability	TCP’s history reflects its adaptability for different cultural contexts
Cab Process	Outer Setting	Partnerships and Connections	Leveraging partnerships and resources from other institutions
		Financing	Navigating Funding Constraints Through Collaborative Problem-Solving
	Inner Setting	Communication	Monthly CAB meetings to ensure consistent communication
		Available Resources	Leveraging organization resources to support program delivery
Cab Outcomes	Implementation	Adaptation of TCP delivery	General tailoring of marketing materials to meet communities’ needs
			Adaptation of TCP delivery for Latinx community
			Adaptation of TCP delivery for AA/Black community
			Adaptations made to the TCP leader certification process
		Dissemination of TCP	Recruitment of TCP class leaders
			Recruitment of community TCP participants
		Reflection and Evaluation	Facilitators of CAB engagement: Acknowledgement of CAB members’ expertise and co-ownership of project Prioritizing community needs alongside research needs Provision of additional resources to support TCP delivery post-study completion
			Barriers to CAB engagement Insufficient funds to support all aspects of the project Initial reluctance by community members while recruiting trainees and community participants Extra workload navigating payment for different entities and individuals Limited resources in community-based organizations
TCP = Tai Chi Prime			

**Table 2:** Overview of perceived barriers and facilitators of CAB engagement and outcomes.

Themes	Subthemes	Quotes
Facilitators of CAB Engagement	Acknowledgement of CAB members’ expertise and co-ownership of project	“If you’re working with these organizations, you have to involve them when you’re writing the grant. You have to really listen carefully, [and] take what they’re willing to teach you. You just have to be humble [and] recognize how little you know.”—Co-PI “…as a community partner, there is just this unconscious inferiority because it’s academia and community-based partners. I think that’s really important when the researchers make all parties at the table feel really comfortable with their role, with their capacity, with what they bring to the table…that’s really important. I think that was demonstrated here.”—CAB 3
	Prioritization of community needs alongside research needs	“I thought this was an amazing team and leadership…it was great opportunity to work and from beginning to end see how we were able to talk about what the community needs were, what the program needs were and blending that well.”—CAB 1
	Provision of additional resources to support program post-study completion	“We have funds left in the [grant] to help support the advancement of teaching skills among the new Tai Chi Prime leaders. We agreed to $30 per session going forward.”—ABM #21,March 2024 “We (researchers) encourage our Milwaukee Community Partner leaders, and the Tai Chi Prime leader representatives to reach out for grant writing assistance.”—ABM #22,April 2024
Barriers to CAB Engagement	Insufficient funds to support all aspects of the project	“[The research team] has discussed a few places where we could trim some budget, but this might take some creativity from all of us.”—ABM #0 (kick-off meeting)—June 2022
	Initial reluctance by community members while recruiting trainees and community participants	“There are misunderstandings about Eastern medicine in some communities, particularly in Christian-based communities. Some of the older generations may not be so familiar, so they don’t quite understand what the movements represent.”—CAB 3
	Extra workload navigating payment for different entities and individuals	“[PI] has had some difficulty in getting all the paperwork filed related to the various payee components of the grant. Extra PO work had to be done because some groups on this team are being paid more than $5000 given their roles and this required extra justifications.” ABM #5,November 2022
	Limited resources in community-based organizations	“If we had more capacity, we would call people [to remind them of class] if we could, but we don’t.”—CAB 1 “As a community-based, organic, small emerging organization, it’s been a little bit of a learning curve for us trying to manage all of the responsibilities of the day-to-day operations while also managing turnover. I speak to that because it is impeding our ability to have consistent invoicing for the project.”—CAB 3

## Data Availability

The data generated and analyzed in this study are not publicly available due to the nature of the data (i.e., participants’ personal experiences). However, access to the data (in anonymized form) may be granted from the corresponding author upon reasonable request.

## Abbreviations

The following abbreviations are used in this manuscript:

CBPR	Community-Based Participatory Research
CAB	Community Advisory Board
TCP	Tai Chi Prime
CFIR	Consolidated Framework for Implementation Research

## Appendix A

**Table A1:** Description of the CAB Membership.

PARTNER ORGANIZATIONS	ORGANIZATION DESCRIPTION	INDIVIDUALS NOMINATED TO SERVE ON CAB
**CORE EL CENTRO**	A Milwaukee-based non-profit organization that provides affordable natural healing and wellness services such as acupuncture, yoga, dance, cooking and herbalism workshops to people with little to no healthcare access. Classes are offered in English and Spanish.	Two individuals (one Executive Director and one staff health navigator) who also enrolled in the Tai Chi Prime leader training
**UNITE WI**	A non-profit that primarily serves the Milwaukee AA/Black community. The organization connects Community Health Workers (CHW) to people needing well-being programs, healthcare services, and social services.	One Executive Director
**WALNUT WAY**	Non-profit catering primarily to AA/Black and low-income families in Milwaukee. The organization offers various activities and programs to support community development, including wellness programs.	Two (one Grant Manager and one yoga instructor/Tai Chi Prime leader training candidate)
**TAI CHI HEALTH**	Purveyor of Tai Chi Prime. The organization offers several tai chi programs through its network of certified Yang-style trainers. Since its inception almost 50 years ago, the organization has trained over 10,000 students, including physical and occupational therapists.	Two (one Executive Director and one Spanish-speaking tai chi instructor)
**ENHANCING BALANCE**	Milwaukee-based tai chi and Tai Chi Prime instructor. Senior master trainer certified to train and assess fidelity of TCP leaders.	One Tai Chi Prime Master Trainer

## Appendix B

**Interview Guide for Program Developers (Community Partners, Researchers and Master Trainers)**.

Introduction

Thank you for agreeing to be interviewed today. My name is _____. I am a graduate student at the University of Wisconsin-Madison School of Pharmacy. Today we will discuss your experience developing and implementing the Tai Chi Prime (TCP) leader training course. We would also like your thoughts on challenges with recruitment, training and program adaptations and strategies for overcoming them in future classes. There are no right or wrong answers. Before we begin, do you have any questions you would like to ask about the project? I want to confirm your consent to participate in this research. Is that all right? You can skip any question you don’t want to answer.

If it is OK with you, I will record our discussion today, so I do not miss any important comments. All your comments will be strictly confidential, and if we write any reports or publications about this study, we will not use your name. Would that be all right with you?

1. Please introduce yourself and your role in your organization.
2. Please describe your role in this project.
3. Can you share details about your qualifications, training, and experience in teaching Tai Chi?
4. What inspired you to introduce Tai Chi Prime to African American and Latinx communities?
5. How did you get community buy-in for this project?
6. What strategies did you employ to promote the training courses and generate interest in the community?

Probe: Did you collaborate with any internal departments or external partners to promote the Tai Chi program? If so, how did those collaborations enhance recruitment efforts?

7. How did you tailor your recruitment efforts to appeal to a diverse range of participants with varying levels of familiarity with Tai Chi?
8. What qualifications or criteria did you use to select trainees for the leader pathway training?
9. Can you share any anecdotes or stories that highlight your experience planning and implementing this program?
10. How have you encouraged ongoing participation and engagement in the leader pathway training program
11. Have you observed any particular challenges or barriers to participant retention? If yes, how will you address these issues in the future? If not, why do you think that is?
12. How did you determine the appropriate class duration and frequency for optimal learning and practice?
13. What considerations were made in selecting the class location and setting?

Probe: Could you describe the process of securing and managing the physical space or venue where the Tai Chi classes take place?

14. As part of the advisory board, what kinds of cultural adaptations did your group make to make it more comfortable for the AA/Latinx community.
15. For master trainer: Can you provide insights into how you manage class size and ensure individual attention and guidance for participants?
16. What strategies or modifications did you make during the training to meet the needs of the participants?

Probe: What decisions were made to adapt the training program to meet the cultural, spiritual, and language needs of the two communities?

17. What are your future plans for TCP?
18. What do you need from the research team to support your efforts to get more funding from govt or policy change?
19. What measures are in place to support trainers in offering tai chi prime to their communities in the future?
20. What advice would you give to others considering implementing a falls-prevention program such as Tai Chi Prime in African American/Black and Latinx communities?

CLOSING QUESTION

Thank you very much for sharing your thoughts and experiences. Before we close, is there anything else you want to share about your experience planning and implementing the prime leader training and community Tai Chi Prime?

If you have any questions after this session, please contact me at ______.
